# Different types of low back pain in relation to pre- and post-natal maternal depressive symptoms

**DOI:** 10.1186/s12884-020-03139-9

**Published:** 2020-09-22

**Authors:** Gong Long, Zhang Yao Yao, Yang Na, Yi Ping, Sun Wei, Tan Mingsheng

**Affiliations:** 1 Department of Orthopedic, Friendship Hospital, Peking Union Medica College, Chinese Academy of Medical College, Beijing, China; 2 Department of Obstetrics and Gynecology, West China Second University Hospital of Sichuan University, 610041 Chengdu, Sichuan China; 3 Key Laboratory of Birth Defects and Related Diseases of Women and Children of the Ministry of Education, 610041 Chengdu, Sichuan China; 4 Bao Ding Maternal and Children Hospital, 071000 Baoding, Hebei China

**Keywords:** Low back pain, pregnant women, depression, lumbar pain, posterior pelvic pain

## Abstract

**Background:**

Low back pain (LBP) is a common musculoskeletal problem during pregnancy, with an estimated prevalence ranging from 30–78% (Mota MJ et al. J Back Musculoskelet Rehabil 28(2):351-7,2015 and Abebe E et al. J Med Sc Tech 3(3). 37-44,2014). Women reporting LBP are at increased risk of developing perinatal depression. Pregnancy-related LBP is highly heterogeneous and can be divided into lumbar pain (LP), posterior pelvic pain (PPP), and combined pain (CP). Therefore, the purpose of this study was to investigate the associations between LBP and perinatal depressive symptoms.

**Methods:**

This was a retrospective case-control study conducted from January 2016 to April 2019. A total of 484 pregnant women were enrolled in this study: a case group of 242 pregnant women who were diagnosed with LBP and an age-matched control group of 242 pregnant women without LBP. The Edinburgh Postnatal Depression Scale (EPDS), LBP characteristics, and questionnaires about pregnancy that included demographic, parity, work, comorbidity, and previous pregnancy data were completed and compared between the case group and the control group.

**Results:**

A total of 68 of 242 (28.1%) women experienced PPP, 142 (58.7%) had lumbar pain(LP), and 32 (13.2%) had combined pain. Furthermore, 26.5% of women with prenatal depression in the LP subgroup remained depressed 6 months postnatally, while the percentages for women in the PPP subgroup and CP subgroup were just 10.6% and 15.6%, respectively. The percentage of women who recovered anytime between delivery and six months postnatally in the PPP subgroup was significantly higher than that in the LP subgroup (31.7% vs. 14.7%, *P* < 0.001).

**Conclusions:**

There is a difference in the prevalence of prenatal, postnatal, and perinatal depressive symptoms among pregnant women with different types of LBP. It is necessary to screen prenatal and postnatal depression separately and differentiate the types of LBP during pregnancy. Attention to these factors may help to outline better management strategies to improve maternal health.

## Background

Low back pain (LBP) is a common musculoskeletal problem during pregnancy, with an estimated prevalence ranging from 30–78% [1, 2]. This condition is generally severe enough to interfere with daily life, causing limitations in performance and productivity at work [3]. The consequences of pregnancy-related LBP involve increased sick leave, high rates of functional disability, and increased seeking of treatment for symptom relief. However, regression of LBP after delivery may be slow and incomplete, and 15% of women with this condition have dated the commencement of pain to the time of one of their pregnancies [4]. These intractable reactions may trigger perinatal depression [5].

Perinatal depression is defined as prepartum depression lasting up to 1 year postpartum. Perinatal depression is common morbidity during pregnancy and lactation, with international prevalence rates ranging between 8% and 36% [6], and this disease compromises maternal and even paediatric health [5, 6]. The aetiology of perinatal depression is multifactorial and complex. Many psychological, psychosocial, socioeconomic, and obstetric risk factors, such as educational level, annual household income, and unexpected sex of the baby, have been reported to be associated with this mental disorder [6, 7]. Women reporting LBP are at increased risk of developing perinatal depression [6, 8, 9].

Previous studies have concurred that pregnancy-related LBP is highly heterogeneous and can be divided into lumbar pain (LP) and posterior pelvic pain (PPP) [8, 10–14]. The former is rather constant throughout pregnancy with a frequency of approximately 10%, whereas the latter seems to increase in frequency in the beginning and remain constant at a higher level, approximately 35% throughout pregnancy [10]. After delivery, regression of the different pain types also differs substantially. Lumbar pain does not regress as expected, whereas posterior pelvic pain diminishes in week 11 postpartum to approximately 5% [11].

Perinatal depression can be divided into prepartum depression and postpartum depression by the event of delivery. Just because of this event, different LBP subtypes began developing toward different directions [11]. At present, the association between LBP and depression has been well known to clinicians [5, 6]. However, such an association is overly general and lacks further deep and specific insight. In the clinic, a pregnant woman with LBP should be determined to type her pain before offering the appropriate treatments. Besides, if depression is present for this woman at the same time and when it occurs (preoperative, postoperative, or both) should also be considered. Therefore, it is far from taking further and individualized measures to deal with these two conditions for clinicians with a superficial understanding of their connections. To our best knowledge, there are few studies about the characteristics of perinatal depression across LBP subtypes and their associations. Therefore, the purpose of this study was to investigate the association between LBP and perinatal depression and the characteristics of depressive symptoms among pregnant women with different types of LBP.

## Methods

### Design

This was a retrospective case-control study conducted from January 2016 to April 2019. This research has been approved by the IRB of the authors’ affiliated institutions.

### Subjects

All the enrolled women who attended the antenatal clinic in the Department of Gynecology, University Hospital of ** gave written consent. The pregnant women who were diagnosed with LBP were assigned as the case group, and the age-matched healthy pregnant women without LBP were assigned as the control group from the same hospital during the same time. Pregnant women generally need to register at an obstetrics unit in the 12th week of pregnancy. They are examined due to obstetric reasons on 12–14 scheduled dates during the whole pregnancy. Nineteen women with a history of any disease before pregnancy or substance abuse were excluded. Another 32 women also had to be excluded: lost to follow-up/incomplete data (N = 09), feelings of severe and constant fatigue(N = 00), adverse life events during pregnancy and previous pregnancy including unplanned abortion, severe foetal malformations, and dead foetuses due to potential risks for perinatal depression(N = 07) [5], pregnancy via reproductive medicine (N = 01), loss of close companions/family members/friends during the previous 12 months (N = 08), and severe hypertension and diabetes during pregnancy (N = 07). Finally, a total of 484 pregnant women were enrolled in this study: a case group of 242 pregnant women with LBP and an age-matched control group of 242 pregnant women without LBP. The patients in the case group were further divided into three groups: the LP group, PPP group, and CP group.

### Instruments

#### *Edinburgh Postnatal Depression Scale (EPDS)*

**Fig. 1:**
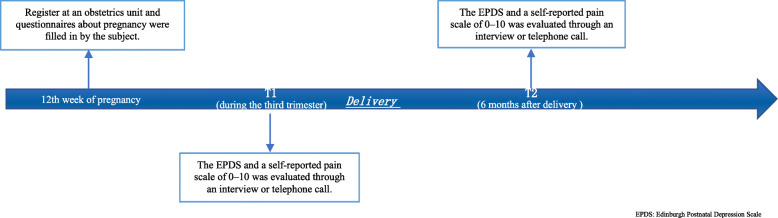
The percentage of different depression status in control group and case subgroups

The outcome of interest was a positive screen for perinatal depression symptoms using the EPDS [15]. Women who have a consultation in an antenatal clinic in our hospital are routinely administered the EDPS to screen for depression. This scale consists of 10 short questions with a choice of four answers that closely reflects how she was feeling over the past seven days. Scores are recorded as 0, 1, 2, and 3 according to symptom severity. Certain question items (i.e., 3, 2, 1, and 0) are scored in a reverse manner. The EPDS has been studied extensively, and it is thought to be a valid screen for both pre- and postnatal depression [15–17]. The EPDS has been widely used for research and for use in the community to screen for pregnancy-related depression with a sensitivity of 86%, a specificity of 78%, and a positive predictive value of 73% [15]. A score ≥ 13 on the EPDS is the recommended cutoff to use for identifying probable major depression perinatally [15]. The EPDS was administered by an experienced psychiatrist through an interview or telephone call. Each woman was evaluated once for this rating in the morning during the third trimester (T1) before delivery and six months (T2) after delivery (Fig. 1). Perinatal depression is represented by a positive screen for both prenatal and postnatal depression [5].

#### *Description of low back pain (LBP) and its subdivisions*

LBP in pregnancy has been defined as a recurrent or continuous pain rating of ≥ 3 for more than one week from the lumbar spine or pelvis [14]. The pain intensity was evaluated with the self-reported scale of 0–10 (0 as no pain to 10 as the worst possible pain) to screen LBP through an interview or telephone call at the same time points as those of the EPDS (Fig. 1). A recurrent or continuous LBP rating of ≥ 3 has a disabling influence on the quality of life [18], and previous studies have demonstrated that disabling LBP has a close association with depression [6, 7]. The exposures of interest were binary variables about the pain types perinatally (lumbar pain = 1 and posterior pelvic pain = 0 during the data input).

Lumbar pain (LP) was characterized by a history of lumbar back pain before pregnancy, pain drawing with markings above the sacrum in the lumbar spine, a decreased range of motion in the lumbar spine, pain upon palpation of the erector spinae muscle and negative results on the posterior pelvic pain provocation test. PPP was characterized by no history of lumbar back pain before pregnancy, pain drawing with markings in the gluteal area, time- and weight-bearing related to pain deep in the gluteal area, pain-free intervals, free range of motion in the spine and positive results on the posterior pelvic pain provocation test[8]. Combined pain was defined as having both LP and PPP.

During the period of pregnancy and six months postnatally, all the women who experienced LBP would be referred to a multidisciplinary team, which included an obstetrician, orthopaedist, acupuncturist, and physiotherapist. This team, the participants of whom were blinded to the results of the depressive evaluation, identified the pain types according to the characteristics mentioned above. According to the results, treatments would be recommended, including education regarding anatomy and kinesiology, back-strengthening exercises, reducing physical activity, avoiding overloading the pelvis, physiotherapy, manipulation, yoga training, and/or acupuncture. The treatment plan depended on the needs of the particular women and the discomfort level [14].

#### *Questionnaire about the pregnancy*

This questionnaire was designed by the authors, and it collected data that included age, BMI, educational level, annual household income, caesarean delivery, breastfeeding, unexpected sex of the baby, parity, sick leave, large amount of physical demand (twisting/lifting movements) and LBP in the previous pregnancy. It was filled in by the subject before the first assessment (Fig. 1). Participants were asked to give their choices. In accordance with the rule of at least ten events per variable in the analysis, the number of variables had to be limited [19].

#### *Sample size*

The sample size was calculated to detect a mean difference in pain scores of 0.5 with a standard deviation (SD) of 0.25. The error was set at 0.05, and the power level was set at 90% with additional compensation for a possible dropout rate of 20%. The required sample size was at least 28 patients in each group.

### Statistical analysis

The Kolmogorov-Smirnov test was used to assess the distribution of continuous variables. According to the results of the Kolmogorov-Smirnov test, we used the mean and standard deviation or the median and semi-interquartile range to demonstrate normally distributed and non-normally distributed variables, respectively. Ordinal variables were described as proportions. The Chi-square test was carried out to compare dichotomous variables, and Student’s t-test was used for continuous variables. The Kruskal-Wallis test was used for multigroup comparisons of nonparametric data on the ordinal level. Logistic regression was performed to estimate the odds ratio (OR) and the associated 95% confidence interval (CI) to determine LBP types in perinatal depression. Multivariate logistic models were performed using stepwise elimination of variables of interest from univariate analysis after adjustment for confounding factors. Logistic regression analysis was used to examine the association between depression pre- and postnatally, different types of LBP, and possible confounders. The dependent variable was depression pre- and postpartum. Different LBP types were entered as categorical independent variables (no LBP as a reference). The covariates were suspected if the prevalence of the LBP type and perinatal depression were higher when the risk factor was present and if the crude odds ratios were statistically significant for each association. The power of the sample size was calculated by G*Power (version 3.1, Heinrich-Heine-Universita¨t Du¨sseldorf, Germany). The statistical significance and power analysis were P-values ≤ 0.05 and 0.8, respectively. SPSS version 22 (SPSS; Chicago, IL, USA) was used to perform all analyses.

## Results

The mean age of the 242 women with LBP and without LBP was 27.4 ± 4.5 years and 27.6 ± 4.8 years, respectively. A total of 68 of 242 (28.1%) women experienced PPP, 142 (58.7%) had lumbar pain, and 32 (13.2%) had combined pain. The clinical data of the four groups classified based on the type of LBP are shown in Table 1. The overall sociodemographic patient characteristics were similar across LBP types, with the exception of “a large amount of physical demand” and “LBP in previous pregnancy” (Table 1).

**Table 1:** Characteristics of the studied women

	Without LBP (*n* = 242)	LP (*n* = 68)	PPP (*n* = 142)	CP (*n* = 32)	*P* value
Age (Mean ± SD) (years)	27.4 ± 4.5	26.8 ± 4.2	27.8 ± 4.7	28.4 ± 3.9	0.297
BMI (Mean ± SD) (kg/m2)	26.0 ± 1.2	26.2 ± 1.3	25.7 ± 1.8	25.8 ± 1.6	0.079
Educational Levels (≥ high school/university) (N,%)	132(54.5%)	32(47.1%)	80(56.3%)	14(43.8%)	0405
Household annual income (Dollars)	1400.5 ± 600.3	1498.5 ± 580.3	1540.5 ± 612.3	1389.5 ± 670.3	0.140
Caesarean delivery (N, %)	26(10.7%)	9(13.2%)	18(12.7%)	6(18.8%)	0.609
Breast-feeding (N, %)	212(87.6%)	58(85.3%)	122(85.9%)	24(75%)	0.290
Unexpected gender of the baby (N, %)	25(10.3%)	9(13.2%)	18 (12.7%)	5(15.6%)	0.755
Primigravida (N, %)	168(69.4%)	48(70.6%)	103(72.5%)	22(68.9%)	0.927
Sick leave ≥ 90 days (N, %)	32(13.2%)	13(19.1%)	23(16.2%)	4(12.5%)	0.610
Large amount of physical demand (Twisting/lifting movements) (N, %)	52(21.5%)	30(44.1%)	34(23.9%)	9(28.1%)	0.002*
LBP in previous pregnancy (N, %)	80(33.1%)	39(57.4%)	76(53.5%)	19(59.4%)	< 0.001*

*P* values from ANOVA, Kruskal-Wallis or Chi test. All original 2-tailed *P* values were multiplied by 6 (Bonferroni correction). *indicates statistically significant. *LBP *Low Back Pain, *LP* Lumbar Pain, *PPP* Posterior Pelvic Pain, *CP* Combined Pain, *SD* Standard Deviation, *BMI* Body Mass Index

### 3.1 The reliability analysis of measurements

The internal reliabilities of the EPDS in this study at both time points were good (Cronbach’s alpha: T1α = 0.835; T2α = 0.826).

### 3.2 Depressive symptoms and different LBP types in pregnant women

Before delivery, the prevalence of depressive symptoms was higher among women with LBP (lumbar pain + posterior pelvic pain + combined pain) than among women without LBP (26.0% vs. 11.2%, *P* < 0.001). Women with PPP had a higher prevalence of depressive symptoms than those without LBP (*P* < 0.001) and those with LP (*P* = 0.014) (Table 2). However, there was no difference in the prevalence of depressive symptoms between those with PPP and those with the remaining LBP types (Fig. 1).

**Table 2:** Crude and adjusted odds ratios (ORs) and 95% confidence intervals (CIs) for the association between LBP type and perinatal depression

Independent Variables	Prenatal Depression		Postnatal Depression		Perinatal Depression	
	Adjusted OR#	P	Adjusted OR#	P	Adjusted OR#	P
LBP types						
No LBP (ref.)						
LP	1.1 (0.7–1.7)	< 0.001*	3.2 (2.1–4.8)	< 0.001*	3.6(3.0-4.3)	< 0.001*
PPP	2.9 (1.7–4.9)	< 0.001*	1.1 (0.6–1.9)	< 0.001*	1.1(0.8–1.5)	< 0.001*
CP	2.3 (1.2–4.6)	< 0.001*	1.9 (1.0-3.8)	< 0.001*	2.1(1.4–3.2)	< 0.001*
Educational level ≥ high school/university	0.4 (0.3–0.6)	< 0.001*	0.4 (0.3–0.6)	< 0.001*	0.4(0.3–0.6)	0.004*
Household annual income (<$6,000)	1.2 (0.7–2.2)	< 0.001*	1.4 (0.8–2.5)	< 0.001*	1.3(0.7–2.3)	< 0.001*
Sick leave ≥ 90 days	1.1 (0.7–1.8)	< 0.001*	1.2 (0.8–1.9)	0.004*	1.5(0.9–2.4)	0.003*
Primigravida	1.3 (0.9-2.0)	< 0.001*	1.4 (0.9–2.1)	< 0.001*	1.4(0.9–2.1)	< 0.001*
Unexpected sex of the baby	1.4 (0.9–2.2)	0.001*	1.3 (0.8–2.1)	0.004*	1.5(0.9–2.4)	0.005*
Normal BMI (18.5–23.9)	1.2 (0.8–1.8)	< 0.001*	1.3 (0.9-2.0)	< 0.001*	1.1(0.7–1.7)	< 0.001*

*indicates statistical significance. # fully adjusted by confounding factors. Odds ratios as well as 95% CIs were shown. *OR* Odds Ratio, *LBP* Low Back Pain, *LP* Lumbar Pain, *PPP* Posterior Pelvic Pain, *CP* Combined Pain, *BMI* Body Mass Index

After delivery, the prevalence of depressive symptoms remained higher among women with LBP than among women without LBP (44% vs. 32%, *P* < 0.001). Women with LP had a higher prevalence of depressive symptoms than those without LBP (*P* = 0.015) and those with PPP (*P* = 0.022) (Fig. 1). However, there was no difference in the prevalence of depressive symptoms between those with LP and those with the remaining LBP types (Fig. 1).

During the perinatal period, the same situation as the postnatal period regarding the prevalence of depressive symptoms was observed (Fig. 1).

### 3.3 Development of depressive symptoms in pregnant women with different LBP types

**Fig. 2:**
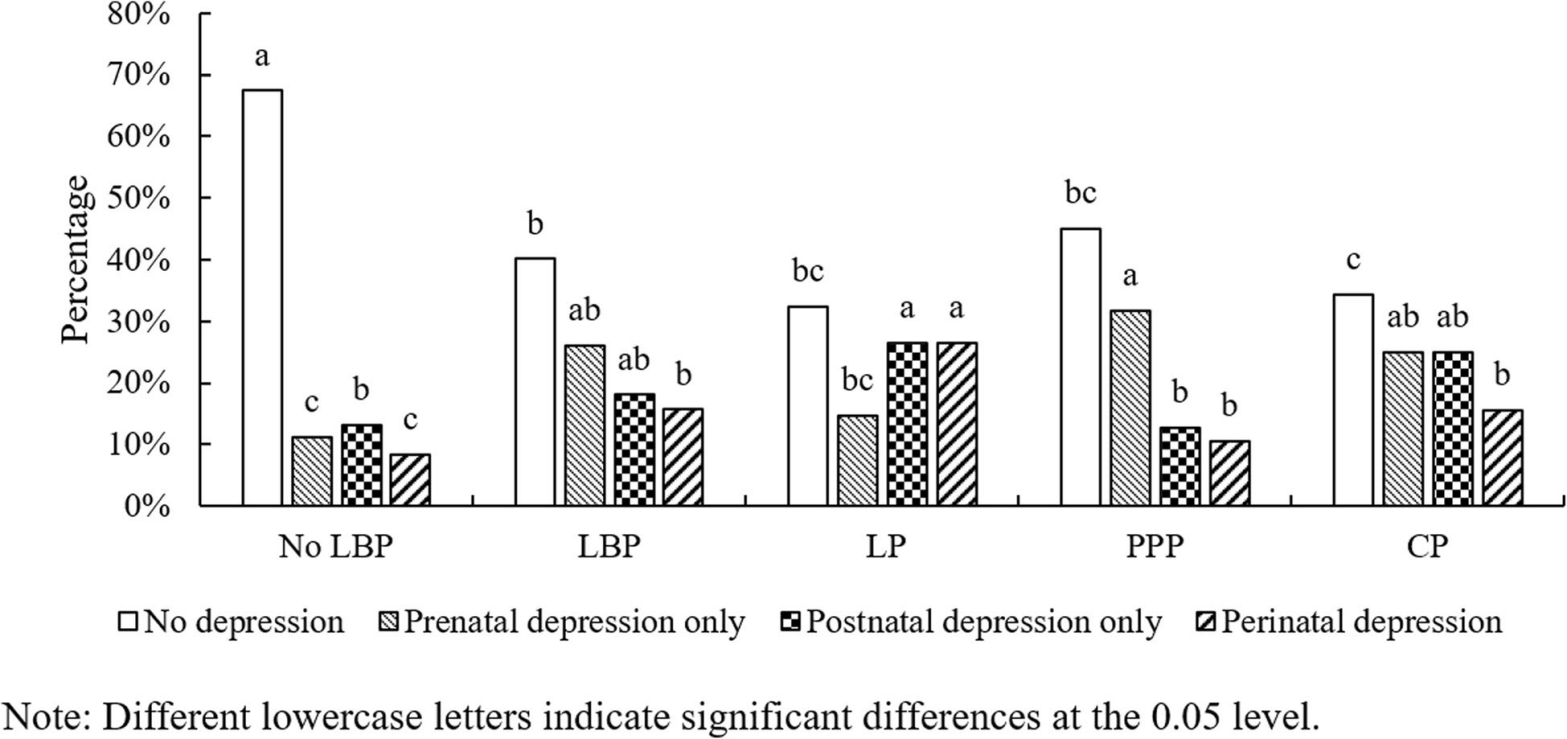
A flowchart about when each measure was administered

The percentage of depressive symptoms in women without LBP slightly increased from the prenatal period (11.2% at T1) to the 6-month postnatal period (13.2% at T2), while depressive symptoms with LBP were reported by more women at T1 (41.7%) than at T2 (33.8%). A decrease in the percentage of women indicating PPP with depressive symptoms was observed after delivery from 31.7–12.7%, while an increase in LP with depressive symptoms was seen from 14.7–26.5%. For CP during the observed period, the percentage of depression remained stable at 25% (Fig. 2).

In the LP subgroup, 26.5% of women with prenatal depression remained depressed at six months postnatally, while this percentage for those women in the PPP subgroup and CP subgroup was 10.6% and 15.6%, respectively. In the PPP subgroup, 12.7% of women who did not have prenatal depression became depressed before six months postnatally, while this percentage for those in the LP subgroup and CP subgroup was 26.5% and 25.0%, respectively. The percentage of women who recovered anytime between delivery and six months postnatally, namely, the percentage of “prenatal depression only,“ in the PPP subgroup was significantly higher than that in the LP subgroup (31.7% vs. 14.7%, *P* < 0.001).

### 3.4 Risk factor analysis for prenatal depression, postnatal depression, and perinatal depression

Prenatal depression was found to have the strongest association with PPP among the three types of LBP after adjusting for the remaining covariates (adjusted OR = 2.9; 95% CI: 1.7–4.9; *P* < 0.001, Table 2).

By contrast, both postnatal depression (OR = 3.7; 95% CI: 2.7–5.6; *P* < 0.001) and perinatal depression (OR = 4.0; 95% CI: 3.3–4.8; *P* < 0.001) were found to have the strongest association with LP after adjusting for the remaining covariates (Table 2).

## 4. Discussion

The symptoms of perinatal depression within our patients included fatigue, anorexia, sleep disorders, interference with affection for the baby, weight loss, and reduced breastfeeding. Our results demonstrated that postnatal depression and perinatal depression were 3.2 times and 3.6 times more prevalent, respectively, in women with LBP than in those without LBP. In contrast, prenatal depression was 2.9 times more prevalent in women with PPP than in those without LBP among all the covariates analyzed in this study. In addition, 26.5% of women with prenatal depression in the LP subgroup remained depressed at six months postnatally, while the percentage for those women in the PPP subgroup and CP subgroup was just 10.6% and 15.6%, respectively. The percentage of women who recovered anytime between delivery and six months postnatally, namely, the figure of “prenatal depression only”, in the PPP subgroup was significantly higher than that in the LP subgroup (31.7% vs. 14.7%, *P* < 0.001). The present study further confirmed the previous finding about the association between LBP and postnatal depression [7–9]. In addition, our results revealed that the prevalence of depression varied across LBP types and fluctuated during the perinatal period.

The aetiology of pregnancy-related depression is multifactorial and complex. Many psychological, psychosocial, socioeconomic, and obstetric risk factors have been reported to be associated with this mental disorder [7]. Patients with well-known risk factors for the onset of pregnancy-related depression were excluded (see exclusion criteria mentioned above) as soon as possible. Among the sociodemographic statistics investigated in this study, we found that low educational level, low income, unexpected sex of the baby, primigravida, and LBP in previous pregnancy were risk factors for the onset of perinatal depression. Of these, LBP in previous pregnancy is first reported. This may be attributed to the fact that repetitive negative thinking may be a key factor in the development and persistence of depression [20]. The remaining risk factors were consistent with previous studies [7, 15, 21].

Many studies have evaluated the association between postnatal depression and LBP [6, 7, 9, 22]. However, few studies have investigated whether there is a difference in the prevalence of depressive symptoms among women with different types of LBP [8]. Although Gutke reported that women with lumbar pain had more depressive symptoms than women without LBP when applying a cutoff score of ≥ 10 or ≥ 13 while women with pelvic girdle pain only screened positive when applying a cutoff of ≥ 10, this study only investigated the prevalence of postnatal depression and compared it across LBP types. Despite strong associations between prenatal and postnatal maternal depression [23], not all pregnant women experience the same course of depressive symptoms, prenatally, or postnatally [24]. This is probably partly due to changes in psychology, physiology, and environment after delivery [24]. LBP, with an estimated prevalence ranging from 30–78% [1, 2], is undoubtedly a common musculoskeletal problem perinatally. However, no study has examined whether a prevalence of incongruence exists due to LBP between pre- and postnatal maternal depression.

The present study showed that prenatal depression was found to have the strongest association with PPP (adjusted OR = 2.9; 95% CI: 1.7–4.9; *P* < 0.001), while both postnatal depression (adjusted OR = 3.2; 95% CI: 2.1–4.8; *P* < 0.001) and perinatal depression (adjusted OR = 3.6; 95% CI: 3.0–4.3; *P* < 0.001) were found to have the strongest association with LP. Due to the retrospective case-control study design, cause and effect cannot be established. Previous studies have shown that patients with LBP are at increased risk for comorbid mental disorders [5–7]. A prospective longitudinal study on depression and chronic musculoskeletal pain suggests a mutually influential relationship between chronic pain and depression [25]. Martini suggested depression to be the logical consequence of having chronic pain [26]. LBP results in the inability to work and social withdrawal, both leading to a feeling of helplessness and despair. Chronic pain is also associated with severe sleep problems and insomnia, both problems that can exacerbate depression and pain [7]. Patients reporting severe pain were far more likely to experience depressive symptoms [6, 7]. The association of chronic pain and depression might be due to shared neurobiological pathways of physical and social pain [25].

Of note, our findings are consistent with Wissart’s results regarding women with LP who had more depressive symptoms than women without LBP when applying a cutoff score of ≥ 10 or ≥ 13, while women with PPP only screened positive when applying a cutoff of ≥ 10. Wissart used two cutoff scores of ≥ 10 and ≥ 13 to screen possible depression, but we did this only with the cutoff of ≥ 13 because this cutoff is more commonly used and indicates more probable depression, which might have been clinically significant depression [27]. Using the cutoff of ≥ 13, the prevalence of depressive symptoms in postnatal women with LBP in our study was 18.1%, which is similar to other studies (14.6%-17%) [6, 8]. By contrast, it was first reported in our study that the prevalence of depressive symptoms in prenatal and perinatal women with LBP was 26% and 15.7%, respectively.

Prenatal depression across LBP types has a different prognosis after delivery. In general, women with depression in the PPP subgroups were nearly twice as likely to have no depression and recovered anytime between delivery and six months postnatally in comparison with those in the LP subgroup (31.7% vs. 14.7%, *P* < 0.001). In addition, women without depression in the LP subgroup and CP subgroup were nearly twice as likely to develop depression before six months postnatally in comparison with those in the PPP subgroup (26.5% vs. 12.7%, 25.0% vs. 12.7%, both *P* < 0.001). While it was clear from the literature at the outset that depression and LBP are indeed related, this study has added to the evidence base by examining the characteristics of depressive symptoms among pregnant women with different types of LBP. Our study supports the dynamic view (from prepartum to postpartum) and multidimensional view (across LBP types) of maternal depressive symptoms among pregnant women. Based on our findings above, it is seemingly crucial for midwives or gynecologists to screen for prenatal and postnatal depression separately and discriminate among the specific types of LBP during pregnancy to identify women at risk and offer appropriate treatment strategies for both symptoms in time. Our results highlight the need to address emotional and physical requirements due to the bio-psychosocial model of pain management. Addressing certain cognitive behaviors, such as functional self-efficacy and catastrophizing behaviors, patients could have less severe pain and less functional disability [28]. Besides, a decrease in the percentage of posterior pelvic pain with depression was observed after delivery, while the reverse situation occurred with lumbar pain. This result helps practitioners give specific advice that women know their possible situation and prognosis about these two conditions before the pregnancy and seek psychological help if necessary.

There were several limitations in our study. First, the primary limitation was its retrospective case-control design, which was less eloquent to deduce causality from the results. Selection bias existed, especially for the possible risk factors for depressive symptoms. Besides, despite the assessment of both pre- and postnatal depression, due to considerations of subject burden, we were limited to assessing depression during the third trimester of pregnancy and at only six months postnatally. Our study did not incorporate potential influences of early maternal behavior and other factors or later postnatal stages, which requires further investigation to cover the whole perinatal period. Finally, our assessment of maternal depressive symptoms was based on a common screening tool designed to elicit a subjective report of mental well-being instead of a clinical diagnosis. These limitations open the door for future studies.

## 5. Conclusion

Prenatal depression was strongly associated with posterior pelvic pain (adjusted OR = 2.9; 95% CI: 1.7–4.9; *P* < 0.001), while postnatal depression (adjusted OR = 3.2; 95% CI: 2.1–4.8; *P* < 0.001) and perinatal depression (adjusted OR = 3.6; 95% CI: 3.0–4.3; *P* < 0.001) were strongly associated with lumbar pain. In addition, a decrease in the percentage of posterior pelvic pain with depression was observed after delivery, while the reverse situation occurred with lumbar pain. It is necessary to screen prenatal and postnatal depression separately and differentiate among LBP types during pregnancy. Attention to these factors may help to outline better management strategies to improve maternal health.

## Data Availability

The datasets generated and/or analysed during the current study are not publicly available due to restrictions associated with anonymity of participants but are available from the corresponding author on reasonable request.
